# Mindfulness and self-rated performance among novice athletes in China: a sequential mediating role of flow and cognitive anxiety

**DOI:** 10.3389/fpsyg.2025.1436321

**Published:** 2025-07-09

**Authors:** Jian Peng, Longjun Jing, Peng Wang, Huilin Wang

**Affiliations:** 1 School of Physical Education, Hunan University of Science and Technology, Xiangtan, China; 2 School of Business, Hunan University of Science and Technology, Xiangtan, China

**Keywords:** novice athletes, mindfulness, flow, cognitive anxiety, self-rated performance

## Abstract

**Purpose:**

This study explores the relationship between mindfulness, flow, cognitive anxiety, and self-rated performance in competitive sports, moving away from traditional cognitive-behavioral principles to investigate mindfulness-based interventions, specifically mindfulness- and acceptance-based interventions (MABI). The research is centered on understanding how mindfulness relates to the self-rated performance, flow experiences, and cognitive anxiety in novice athletes.

**Methods:**

Convenience sampling and snowball sampling were employed to collect cross-sectional data from 315 participants in Changsha, China, during August and September 2023. Utilizing a structural equation model, the study examined the association between mindfulness and the self-rated performance of novice athletes.

**Results:**

Mindfulness, flow, and self-rated performance were all negatively correlated with cognitive anxiety, and cognitive anxiety mediated the relationship between flow and self-rated performance. Additionally, flow and cognitive anxiety served as a chain mediation between mindfulness and self-rated performance.

**Discussion:**

The findings suggest that for novice athletes, flow and cognitive anxiety may be crucial mechanisms through which mindfulness improves self-rated performance. When coaches face novice athletes experiencing suboptimal competitive states, they can utilize effective mindfulness practices to enhance the athletes' perception of their performance. This can help novices transition to elite roles and integrate more quickly into the professional sports arena.

## 1 Introduction

Since the early 1980s, influenced by theories of cognitive psychology, the field of competitive sports has sought to optimize athletes' performance through psychological interventions (Zakrajsek and Blanton, 2017). Interventions have primarily focused on traditional psychological skills training (PST), which involves athletes using learned methods to regulate or enhance their psychological characteristics (Gustafsson et al., 2017). However, research has indicated limitations in the effectiveness of PST in optimizing sports performance. Firstly, only a few studies have revealed the relationship between PST and sports performance. Secondly, the results of numerous studies contradict the foundational assumptions of PST, suggesting that reducing unwanted emotional states or increasing self-confidence does not necessarily lead to enhanced athletic performance (Gardner and Moore, 2004). Lastly, controlling athletes' cognitive processes using traditional PST methods proves difficult (Birrer et al., 2012).

Drawing on the effectiveness of mindfulness- and acceptance-based interventions (MABI) in clinical settings, Gardner and Moore (2004) introduced the mindfulness-acceptance-commitment (MAC) approach, tailored to enhance sports performance. MAC differs from traditional PST as it requires athletes to focus external attention on stimuli relevant to the task at hand, rather than internal processes. It encourages deep engagement in one's sport and a committed pursuit of performance goals. Comparative studies suggest that athletes participating in Athletes undergoing mindful sport performance enhancement (MSPE) training exhibit more pronounced improvements in attention and awareness, as well as greater satisfaction with their performance, in comparison to those undergoing PST (Hut et al., 2023). Consequently, traditional PST interventions, which focus on modifying dysfunctional thoughts and emotions, differ from mindfulness, which prioritizes altering the relationship with physiological and psychological states. Thus, adopting mindfulness appears to be a more effective strategy for optimizing sports performance.

Numerous studies consistently demonstrate a positive relationship between mindfulness and sports performance, particularly in elite athletes (Gross et al., 2018; Wolch et al., 2021). Mindfulness is identified as a key element in improving sports performance by reducing unwanted emotions, clarifying cognitive processes, and enhancing attention to task-related cues (Gardner and Moore, 2004). The research also recognizes the significance of flow concerning mindfulness and athletic achievement. Flow refers to a state of full immersion and focused engagement in the task, facilitated by mindfulness-driven attention and acceptance (Jackson, 2016). Research studies indicate a relationship between mindfulness, optimized sports performance, and a propensity to enter a flow state-a state characterized by seamless, autonomous movement, cognitive clarity, and positive effects.

However, there has been limited attention given to the unique challenges faced by novice athletes—individuals with < 1 year of experience on the team. Unlike elite athletes, novices are often younger and at the beginning stages of their athletic careers. They possess unique characteristics such as insufficient competition experience and high levels of scrutiny and expectations, often leading to pressure from parents and coaches (Brown et al., 2018). Furthermore, when evaluating their performances in elite sports, novice athletes tend to emphasize competition outcomes to impress others or enhance their social status (Patel and Luckstead, 2000). This excessive focus on outcomes can negatively impact their athletic performance, leading to defeats that undermine their confidence, foster self-doubt, and ultimately result in various forms of sports anxiety. To elucidate the complexities of the behavior exhibited by novice athletes, the study adopts the stimuli-organism-response (S-O-R) theory model. In this model, stimuli represent mindfulness practices, the organism signifies the flow state of novice athletes, and the response relates to cognitive anxiety levels and self-rated performance. The study's objectives include examining the interconnections among mindfulness, flow, cognitive anxiety, and self-rated performance in novice athletes, exploring the mediating roles of flow and cognitive anxiety in the relationship between mindfulness and self-rated performance, and providing recommendations for enhancing the perceptual performance of novice athletes.

## 2 S-O-R theory

The S-O-R theory, introduced by Mehrabian and Russell (1974). The S-O-R theory proposes that external environmental stimuli not only lead to a response but also influence internal states in organisms, including aspects such as perception, sensation, and thought. This theory is applicable across various contexts and involves cognitive and emotional systems, taking into account experiences related to long-term memory (Jacoby, 2002).

Mindfulness is a psychological skill that involves paying attention to and accepting present experiences. When combined with the S-O-R theory, it helps individuals better understand and manage their emotional, cognitive, and behavioral responses, thereby processing stimuli, organizing experiences, and selecting responses more consciously. The S-O-R theory is widely recognized in the context of mindfulness and has served as the foundation for Moqbel (2020) exploration of the links between smartphone addiction, mindfulness, wellbeing, health, and technostress among college students. Xiao (2022) elucidated that adolescents, due to their unique developmental stage, display unstable emotional and psychological characteristics. Instances of rejection from family members may diminish mindfulness, leading to unwanted emotions and ultimately resulting in detrimental behaviors, such as excessive reliance on social networks. Gabriella et al. (2021) introduced a model elucidating consumer behavior, suggesting that in the context of second-hand product consumption, electronic word of mouth serves as the central catalyst prompting consumers to participate in mindful consumption behavior. Ben Haobin et al. (2021) identified consumer mindfulness as the psychological mechanism through which a hotel's servicescape influences brand experience, noting that the length of stay negatively moderates this impact.

Building upon the aforementioned research findings, this study's objective is to investigate the connections among mindfulness, flow, cognitive anxiety, and self-rated performance. This exploration aims to enhance the comprehension of the behavior exhibited by novice athletes, ultimately facilitating their rapid progression from novice status to elite levels in their sports careers.

### 2.1 Mindfulness, flow, and cognitive anxiety

Mindfulness is characterized by an individual's deliberate, non-judgmental, and purposeful attention to present experiences (Bishop et al., 2004). Flow refers to a state of complete absorption in an activity, marked by an enhanced sense of creativity and optimal experience (Csimathkszentmihalyi, 2000). Considering the shared characteristics of both mindfulness and flow, particularly their emphasis on wholehearted engagement in the present moment, there exists inherent consistency between the two to some extent. Notably, Kee and Wang (2008) observed a positive correlation between increased mindfulness and higher scores in these flow dimensions.

Examining the positive impact of mindfulness on flow from a theoretical perspective, mindfulness is theorized to assist individuals in directing their attention to the present moment, consequently improving body awareness and emotional regulation. On the other hand, flow involves heightened awareness or emotions achieved by concentrating on a task to improve an individual's optimal performance (Habe et al., 2019). Both share the common emphasis on the importance of being present. Moreover, from the literature, research has shown that mindfulness increases athletes' experiences of flow (Chen and Meggs, 2021; De Oliveira et al., 2024).

The multidimensional theory of competitive anxiety in sport identifies three components: cognitive anxiety, somatic anxiety, and self-confidence, with cognitive anxiety originating from negative expectations and self-evaluations related to success. It involves repetitive thoughts about whether one can or cannot achieve the expected results, difficulties, and an inability to maintain focused attention (Martens et al., 1990). As a focused, non-judgmental awareness of present-moment experiences, mindfulness facilitates engagement with all affective states (positive, negative, and neutral), thereby reducing distress and enhancing cognitive functioning (Dana et al., 2022). Furthermore, studies indicate that mindfulness aids athletes in effectively managing internal responses, including negative self-evaluations, by fostering an attitude of objectively observing their reactions instead of attempting to control them (Ljótsson et al., 2010). Gardner and Moore (2004) demonstrated that highly mindful athletes exhibit enhanced focus on task-relevant thoughts and behaviors while reducing distractibility from external stimuli, somatic sensations, and emotional-cognitive responses. As a result, mindfulness may contribute to a reduction in the occurrence of cognitive anxiety.

Anxiety is a psychological emotion marked by inner turmoil and a sense of fear concerning anticipated events, often viewed as a hindrance to achieving flow states (Csimathkszentmihalyi, 2000). Jackson et al. (1998) have emphasized that the cognitive aspects of anxiety, such as lack of concentration and worries, play a more significant role in impeding flow states compared to physiological aspects. With this in mind, we posit the following hypotheses:

**Hypothesis 1 (H1):** *There is a negative relationship between mindfulness and cognitive anxiety*.
**Hypothesis 2 (H2):** *There is a negative relationship between flow and cognitive anxiety*.

### 2.2 Flow, cognitive anxiety, and self-rated performance

In sports psychology, flow represents an optimal mental state associated with enhanced sports performance through meaningful experiential engagement. It is often described as a potential peak or enhancer of sports performance. Furthermore, the relationship between flow and factors such as focus, motivation, and confidence has been widely studied (Landhäußer and Keller, 2012). Swann et al. (2017) describe the pathways through which flow enhances sports performance as clutch or making it happen. “Clutch” denotes any performance improvement or superior performance observed under competitive pressure, while “making it happen” refers to an individual being in a state more focused on achieving preset goals and current tasks. In the context of athletes, attaining heightened concentration and the seamless integration of action and awareness signify the maximization of flow, leading to the optimization of sports performance. Therefore, athletes are expected to demonstrate better sports performance in a state of flow.

Catastrophe theory suggests that athletes can exhibit optimal sports performance only when cognitive anxiety levels are low. Physiological arousal functions as a mediating variable between cognitive anxiety and sports performance outcomes (Balyan et al., 2016). Moreover, Theories of Attention propose a negative relationship between cognitive anxiety and sports performance. This implies that an individual's cognitive resources are consumed by thoughts of worry and doubt, hindering their utilization for the task at hand (Wine, 1971). Relevant studies have found that athletes, due to their desire for victory and the frequent competition for team rankings, may experience excessive anxiety, frustration, conflict, and fear, especially as cognitive anxiety levels increase, affecting their mental health and sports performance (Parnabas et al., 2015). In summary, the optimization of athletes' sports performance hinges on high levels of flow experience and low levels of cognitive anxiety. Building upon this, the following hypothesis is proposed:

**Hypothesis 3 (H3):** *There is a negative relationship between cognitive anxiety and self-rated performance*.

### 2.3 The mediating effects of flow and cognitive anxiety

Empirical evidence demonstrates that mindfulness can directly promote the generation of flow experiences and can also influence flow experiences by reducing sports anxiety and pessimistic thoughts related to sports. In other words, the higher the level of adherence to and participation in mindfulness practices, the more increased flow experiences and the greater reduction in anxiety and pessimism (Scott-Hamilton and Schutte, 2016). Moreover, from the perspective of mindfulness, the prerequisite for the generation of flow experiences is optimal attention and emotional self-regulation, but this is hindered by high levels of anxiety arousal. This anxiety arousal can lead to a negative self-awareness focus, disrupting concentration and ultimately reducing the likelihood of flow experiences. Jackson et al. (1998) demonstrated that anxiety produced by athletes and perceived skill deficiencies interfere with the generation of flow experiences. They argue that cognitive aspects of anxiety (diverted attention and worrisome thoughts) are the most significant barriers to flow states, ultimately impacting athletes' sports performance.

Following the S-O-R theory, mindfulness serves as a stimulus, and flow reflects the organism's psychological state. Concurrently, cognitive anxiety and self-rated performance emerge as responses displayed by novice athletes. This theory suggests that stimuli have the capacity to directly induce specific behaviors in individuals, and conversely, behavior can reciprocally affect individuals and behavior (Skinner, 1935). Catastrophe theory and Theories of Attention propose that cognitive anxiety in the anxiety dimension is a primary factor influencing athletes' flow experiences (Wine, 1971; Balyan et al., 2016). Therefore, in turn, the organism's flow experiences can also improve and reduce cognitive anxiety. As mindfulness fosters the development of flow experiences, the organism's flow could potentially act as a mediator in connecting mindfulness and cognitive anxiety. Additionally, given that flow experiences form the basis for athletes demonstrating high sports performance, cognitive anxiety might serve as an intermediary variable between organism flow and self-rated performance. In essence, if these assumptions hold true, flow and cognitive anxiety form a chain of intermediaries influencing the relationship between mindfulness and self-rated performance. Based on this, we posit the following hypotheses:

**Hypothesis 4 (H4):** *Cognitive anxiety mediates the relationship between flow and self-rated performance*.
**Hypothesis 5 (H5):** *Flow and cognitive anxiety jointly mediate the relationship between mindfulness and self-rated performance*.

**Figure 1:**
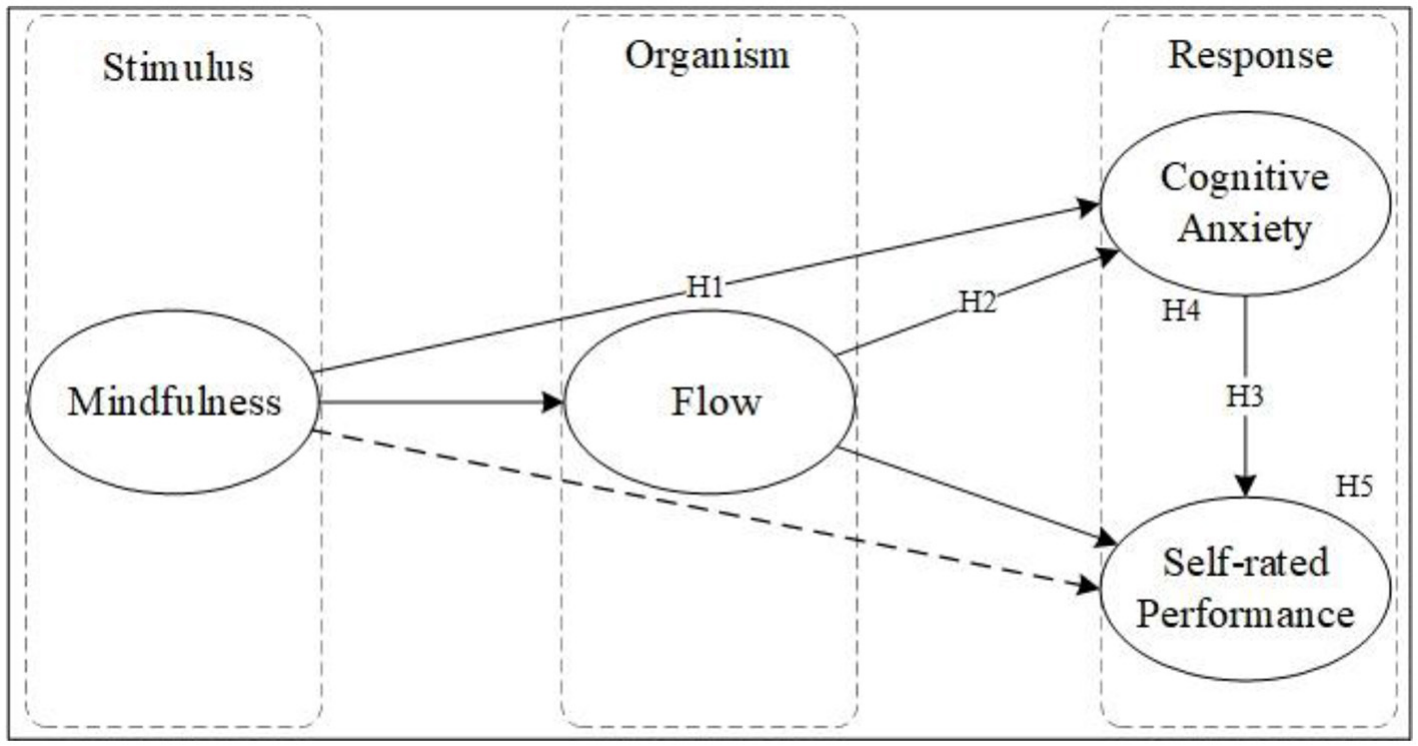
Conceptual framework.

All hypotheses are summarized in Figure 1.

## 3 Methods

### 3.1 Participants

Table 1 presents the demographic characteristics of the 315 novice athletes surveyed, with the following distributions: (1) 67.3% of the participants were aged between 18 and 19. (2) 64.1% were male (*n* = 202), and 35.9% were female (*n* = 113). (3) Regarding competitive experience, 66.0% (*n* = 208) had participated in academy -level competitions, 20.0% (*n* = 63) in regional-level competitions, and 14.0% (*n* = 44) in club-level competitions. (4) In terms of team membership duration, 11.4% (*n* = 36) had joined within the past 6 months, 27.3% (*n* = 86) within 6–9 months, and 61.3% (*n* = 193) within 9–12 months. (5) The athletes were categorized into basketball players (*n* = 125, 39.7%), soccer players (*n* = 103, 32.7%), and volleyball players (*n* = 87, 27.3%).

**Table 1:** Overview of participants (*N* = 315).

**Profiles**	**Survey (%)**
**Age**	
18–19	212 (67.3%)
20–21	73 (23.2%)
≥22	30 (9.5%)
**Gender**	
Male	202 (64.1%)
Female	113 (35.9%)
**Competition level**	
Academy	208 (66%)
Regional	63 (20%)
Club	44 (14%)
**Entry time**	
0 months < T ≤ 6 months	36 (11.4%)
6 months < T ≤ 9 months	86 (27.3%)
9 months < T ≤ 12 months	193 (61.3%)
**Sports items**	
Basketball	125 (39.7%)
Soccer	103 (32.7%)
Volleyball	87 (27.6%)

### 3.2 Procedure

This study concentrated on novice athletes participating in Academy-level and higher-level events, employing a convenient snowball sampling approach. Participants were determined based on the following inclusion and exclusion criteria: (1) Universities with high-level basketball, soccer, and volleyball teams and city-level or higher basketball, soccer, and volleyball clubs were included. (2) Athletes aged 18–24 years with less than 12 months of training were included. (3) Athletes with injuries preventing them from participating in regular training were excluded.

From August to September 2023, researchers surveyed novice athletes from 12 university teams and 20 clubs in Changsha, China. The participants completed questionnaires covering mindfulness, flow, cognitive anxiety, and self-rated performance (18 items). Researchers ensured the confidentiality of responses and addressed any questions from participants throughout the process, with the survey taking ~20 min to complete.

The study received approval from the Ethics Committee of the School of Physical Education, Hunan University of Science and Technology, on March 9, 2023 (No. ECBPEHNUST 2023/003). Participation was voluntary, and participants signed informed consent forms. Out of 400 distributed surveys, 315 valid responses were collected, achieving a response rate of 78.8%.

### 3.3 Instruments

The questionnaire comprises five sections. The first gathers demographic details, primarily including age, gender, level of competition, duration of team training, and type of sport. The second uses the Mindfulness Inventory for Sport scale (Thienot et al., 2014), with items such as “I am able to notice the intensity of nervousness in my body.” The third employs the Dispositional Flow Scale-2 (Jackson and Eklund, 2002; Jackson et al., 2010) for flow state data, with items such as “Challenge-skill balance: I am challenged, but I believe my skills will allow me to meet the challenge.” The fourth assesses cognitive anxiety with the Competitive State Anxiety Inventory-2 (Lane et al., 1999), including items like “I am concerned about performing poorly.” The fifth collects self-rated performance data using items from the Self-rated Performance scale (Tingaz et al., 2023), with items such as “I was very satisfied with my last exercise performance.” All scales use a five-point Likert scale.

To confirm reliability, a pilot test followed guidelines by Kimberlin and Winterstein (2008), yielding 73 valid questionnaires. Results showed all Cronbach's alpha coefficients surpassed 0.8, affirming the adjustments made to the scales.

### 3.4 Data analysis

In this study, we used AMOS v26 to construct a structural equation model (SEM) exploring how novice athletes enhance self-rated performance through mindfulness. Initial assessment of the questionnaire's reliability and validity showed a robust Cronbach's α coefficient of at least 0.859 for all variables.

We then examined fit indices and path coefficients of the hypothesized model, addressing potential mediating effects. Common method variance (CMV) was considered, but Harman's single-factor test results suggested its absence, with a variance extracted of 43.31%, slightly below the conventional threshold of 50% (Podsakoff et al., 2012).

## 4 Results

### 4.1 Reliability and validity

As depicted in Table 2, Cronbach's α ranged from 0.859 to 0.932, exceeding the recommended threshold of 0.70 (Hair et al., 2012), and all composite reliability (CR) values were above 0.85. Minimum values for factor loadings and average variance extracted (AVE) were 0.776 and 0.645, respectively, both exceeding the recommended threshold of 0.50 (Fornell and Larcker, 1981). Thus, all variables demonstrated notable reliability and discriminant validity. Furthermore, Table 3 illustrates that all correlation coefficients were below the square root of AVE, confirming robust discriminant validity across all variables.

**Table 2:** Assessment of reliability and validity.

**Variables**	**Loadings**	**Cronbach's alpha**	**AVE**	**CR**
**Mindfulness (MIN)**		0.901	0.645	0.901
MIN1	0.833			
MIN2	0.802			
MIN3	0.790			
MIN4	0.793			
MIN5	0.796			
**Flow (FL)**		0.917	0.689	0.917
FL1	0.829			
FL2	0.850			
FL3	0.848			
FL4	0.776			
FL5	0.845			
**Cognitive anxiety (CA)**		0.932	0.733	0.932
CA1	0.855			
CA2	0.859			
CA3	0.851			
CA4	0.857			
CA5	0.859			
**Self-rated performance (SRP)**		0.859	0.674	0.861
SRP1	0.858			
SRP2	0.807			
SRP3	0.796			

**Table 3:** Analysis of discriminant validity.

**Construct**	**MIN**	**FL**	**CA**	**SRP**
MIN	**0.803**			
FL	0.432^**^	**0.830**		
CA	−0.439^**^	−0.517^**^	**0.856**	
SRP	0.406^**^	0.571^**^	−0.505^**^	**0.821**

The diagonals (bold) in the matrix represent the square root of the average variance extracted (AVE), while off diagonals display Pearson's correlation coefficients between variables. ^**^p < 0.01. MIN, Mindfulness; FL, Flow; CA, Cognitive Anxiety; SRP, Self-rated Performance.

### 4.2 Structural path model

**Figure 2:**
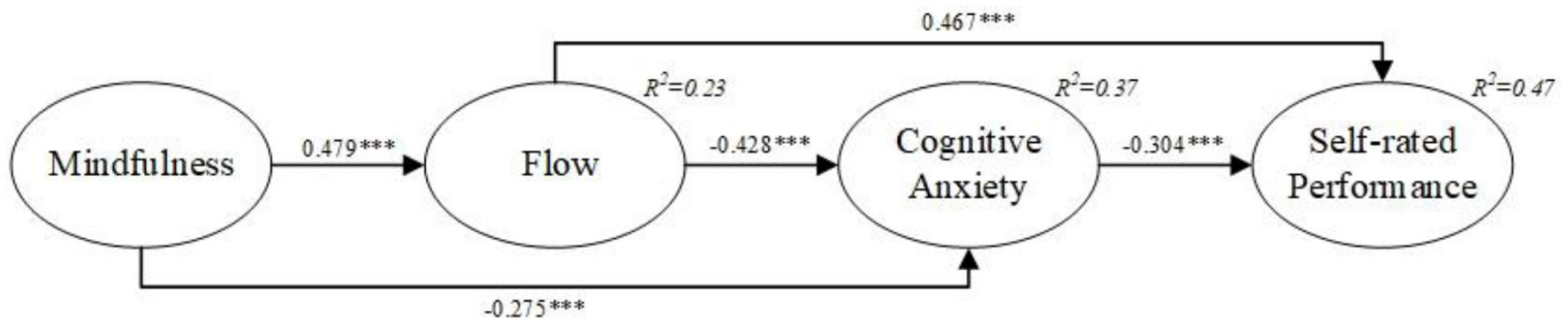
Structural model. ***p < 0.001.

Initially, the fit indices, aligning with prior studies (Jackson et al., 2009; Hair et al., 2012) recommendations, affirm a favorable fit for the data (χ^2^/df = 1.259, GFI = 0.946, NFI = 0.960, CFI = 0.992, TLI = 0.990, IFI = 0.992, RMSEA = 0.029) and the structural model. Second, Pearson correlation results in Table 3 reveal significant associations among independent, mediating, and dependent variables, supporting hypothesis validation. Lastly, the structural path model in Figure 2 shows statistically significant relationships: mindfulness and cognitive anxiety (β = −0.275, *p* < 0.001), confirming Hypothesis 1 that mindfulness is negatively correlated with cognitive anxiety. Flow and cognitive anxiety (β = −0.428, *p* < 0.001), confirming Hypothesis 2 that flow is negatively correlated with cognitive anxiety. Cognitive anxiety and self-rated performance (β = −0.304, *p* < 0.001), confirming Hypothesis 3 that cognitive anxiety is negatively correlated with self-rated performance.

### 4.3 Mediation test

Researchers proposed that cognitive anxiety mediates between flow and self-rated performance, and flow and cognitive anxiety act sequentially as mediators between mindfulness and self-rated performance. To test these mediations, the study followed Bollen and Stine (1990) suggestion, employing the bootstrap method with 5,000 bootstrap samples for 95% confidence intervals. Results in Table 4 show *Z*-values exceeding 1.96, with zero not within the intervals. This indicates significant mediation: cognitive anxiety mediates between flow and self-rated performance (β = 0.130, *p* < 0.001), confirming Hypothesis 4 that cognitive anxiety mediates the relationship between flow and self-rated performance. Additionally, flow and cognitive anxiety serve as sequential mediators between mindfulness and self-rated performance (β = 0.370, *p* < 0.01), confirming Hypothesis 5 that flow and cognitive anxiety mediate the relationship between mindfulness and self-rated performance. Findings suggest that novice athletes practicing mindfulness exhibit lower cognitive anxiety, higher flow experience, and enhanced self-rated performance.

**Table 4:** Mediation effect testing.

**Path**	**Point estimate**	**Product of coefficients**		**Bootstrapping**		
				**Bias-corrected 95% CI**		**Two-tailed significance**
		***SE***	***Z***	**Lower**	**Upper**	
FL → SRP	0.130	0.035	3.714	0.075	0.214	< 0.001
MIN → SRP	0.370	0.039	9.487	0.293	0.447	< 0.01

## 5 Discussion

### 5.1 Theoretical contributions

The primary objective of this research is to explore the predictive impact of mindfulness on the self-rated performance of novice athletes, specifically examining the mediating influences of flow and cognitive anxiety. Utilizing the S-O-R model as its theoretical framework, the study applies SEM techniques for analysis. The results uncover inverse relationships among mindfulness, flow, and cognitive anxiety, along with a negative relationship between cognitive anxiety and self-rated performance.

These findings align with prior research that highlights the inverse relationship between mindfulness and competitive anxiety (Röthlin et al., 2016), the interplay between flow and anxiety (Koehn, 2013), and the negative impact of anxiety on self-rated performance (Tingaz et al., 2023). A unique contribution of this study lies in its focused exploration of cognitive anxiety as a key psychological mechanism within sports performance. Cognitive anxiety, a critical subcomponent of sports-related anxiety, has long been identified as a significant impediment to achieving flow states (Jackson et al., 1998). Theoretical perspectives such as catastrophe theory (Balyan et al., 2016) and theories of attention (Wine, 1971) further support the view that cognitive anxiety detracts from athletic performance, establishing a negative correlation between the two.

The results also indicate that cognitive anxiety partially mediates the relationship between flow and self-rated performance. This suggests that novice athletes—who are often more emotionally reactive in competitive settings—are especially prone to elevated cognitive anxiety, which, in turn, impairs their ability to perform effectively (Parnabas et al., 2014). Meta-analytical evidence further confirms the negative association between cognitive anxiety and performance, while identifying moderating variables such as gender and competitive experience (Woodman and Hardy, 2003). Given that anxiety reflects internal psychological distress, and cognitive anxiety specifically disrupts attentional focus, its role as a mediator is both theoretically and empirically supported.

Furthermore, the study reveals a sequential mediation pathway in which both flow and cognitive anxiety mediate the relationship between mindfulness and performance. Within the S-O-R framework, flow represents the organismic state influenced by internal anxiety levels. The observed inverse relationship between cognitive anxiety and flow suggests that reducing anxiety through mindfulness may help foster optimal psychological states. Empirical findings from mindfulness intervention studies by (Scott-Hamilton et al. 2016), (Sparks and Ring 2022), and (Röthlin et al. 2016) corroborate this mechanism, showing that mindfulness not only enhances flow experiences but also improves athletic outcomes. By promoting present-moment awareness and non-judgmental acceptance, mindfulness can serve as a psychological buffer, enabling novice athletes to enter flow states more easily and perform better.

### 5.2 Practical implications

This study contributes to the research on optimizing the performance of novice athletes in several ways: Firstly, given the potential limitations of traditional psychological skills training (PST), this study introduces mindfulness as an alternative method for enhancing athletic performance. Rather than relying solely on external evaluations, performance is assessed through athletes' self-ratings, providing a more nuanced, athlete-centered perspective. This methodological shift opens new avenues for evaluating performance in novice athletes and may inform future research and training programs tailored to athletes at different stages of development.

Second, while prior research has primarily emphasized technical training, physical conditioning, and coaching strategies, comparatively less attention has been paid to the role of psychological and situational factors in performance. By focusing on mindfulness, flow, and cognitive anxiety, this study contributes a theoretical foundation for understanding how internal states influence athletic outcomes—specifically through the lens of Mindfulness-Acceptance-Based Interventions (MABI). These findings suggest that psychological readiness is just as critical as physical preparation in the pursuit of athletic excellence.

Lastly, grounded in the S-O-R theoretical model, the study identifies cognitive anxiety as a key factor influencing the emergence of flow states and subsequent performance outcomes. The results indicate a reciprocal relationship: flow experiences help to reduce cognitive anxiety, and lower cognitive anxiety in turn facilitates better performance. Mindfulness serves as a foundational element in this process by fostering the conditions necessary for flow while mitigating anxiety. These interrelationships underscore the importance of cultivating mindfulness as a psychological skill in novice athletes.

In practical terms, mindfulness entails the deliberate, non-judgmental awareness of the present moment. In athletic contexts, this translates to maintaining focused attention on goal-relevant thoughts and actions while minimizing cognitive disruptions from external distractions or emotional fluctuations (Gardner and Moore, 2004). Through reducing maladaptive emotional responses—such as fear of failure or excessive self-doubt—mindfulness enhances both emotional regulation and attentional control. Moreover, theoretical models such as Catastrophe Theory and Attentional Control Theory (Balyan et al., 2016; Wine, 1971) support the view that elevated levels of cognitive anxiety can negatively impact performance and mental health. Mindfulness-based training, which emphasizes present-focused attention and emotional regulation, can mitigate these effects and promote entry into flow states (Chen and Meggs, 2021). The overlap between the attentional demands of mindfulness and flow suggests that mindfulness practices may serve as a gateway to achieving flow, particularly for novice athletes who are more susceptible to psychological instability under competitive stress. Given these findings, coaches should recognize the practical value of mindfulness in sport-specific contexts. They are encouraged to integrate mindfulness-based techniques into training routines, while simultaneously monitoring athletes' psychological and physiological indicators. Intervention strategies should be customized to reflect the individual needs and stress responses of athletes. Likewise, athletes should be guided to develop mindfulness awareness and emotional insight, enabling them to regulate internal distractions such as worry and rumination, which are detrimental to performance.

## 6 Limitations

This study has several limitations that should be acknowledged. First, although it explores associations between mindfulness, flow, cognitive anxiety, and self-rated performance—along with the mediating roles of flow and cognitive anxiety—it does not permit causal inferences due to its correlational design. Second, the study focused exclusively on flow and cognitive anxiety as mediators, potentially overlooking other important psychological factors such as emotion regulation and psychological resilience, which may also influence self-rated performance. Future studies should incorporate a broader range of mediating variables to gain a more comprehensive understanding of the underlying psychological mechanisms. Third, the reliance on self-reported data introduces the possibility of response bias, which may affect the validity of the results. Lastly, as the sample consisted solely of novice athletes, the generalizability of the findings to more experienced or elite athlete populations remains uncertain.

## 7 Conclusion

The distinctive attributes of novice athletes, encompassing their youthful age, limited competitive exposure, and the substantial pressures and expectations they encounter, render them susceptible to unwanted emotions such as self-doubt and worry, ultimately influencing their athletic performance. The S-O-R theoretical model, coupled with mindfulness practices rooted in MABI, promotes the generation of flow states and inhibits the development of cognitive anxiety. This framework lays the groundwork for advancing athletic performance. The study results indicate that cognitive anxiety has a negative impact on mindfulness, flow, and self-rated performance. Additionally, cognitive anxiety mediates the relationship between flow and self-rated performance. Finally, there is a chain mediation effect of flow and cognitive anxiety between mindfulness and self-rated performance.

## Data Availability

The raw data supporting the conclusions of this article will be made available by the authors, without undue reservation.
