# Correction: Mindfulness and self-rated performance among novice athletes in China: a sequential mediating role of flow and cognitive anxiety

**DOI:** 10.3389/fpsyg.2025.1719909

**Published:** 2025-11-10

**Authors:** Jian Peng, Longjun Jing, Peng Wang, Huilin Wang

**Affiliations:** 1 School of Physical Education, Hunan University of Science and Technology, Xiangtan, China; 2 School of Business, Hunan University of Science and Technology, Xiangtan, China

**Keywords:** novice athletes, mindfulness, flow, cognitive anxiety, self-rated performance

The reference for the Dispositional Flow Scale-2 (DFS-2) was erroneously written as Gouveia M. J. P. M., Ribeiro J. L. P., Marques M. M., Carvalho C. M. C. F. D. (2012). Validity and reliability of the Portuguese Version of the Dispositional Flow Scale-2 in exercise. *Rev. Psicol. Deporte* 21, 81–88. Available online at: https://www.redalyc.org/articulo.oa?id=235124455011

It should be Jackson, S. A., and Eklund, R. C. (2002). Assessing flow in physical activity: the Flow State Scale-2 and Dispositional Flow Scale-2. *J. Sport Exerc. Psychol*. 24, 133–150. doi: 10.1123/jsep.24.2.133; Jackson, S. A., Eklund, R. C., and Martin, A. J. (2010). *The FLOW Manual. Mind Garden Inc*. Available online at: https://www.mindgarden.com/100-flow-scales (Accessed October 6, 2025).

The reference above (Gouveia et al., 2012) was cited in the manuscript in place of the original DFS-2 citations, in the **Methods** section, 3.3 *Instruments*. This sentence previously stated:

“The third employs the Dispositional Flow Scale-2 (Gouveia et al., 2012) for flow state data.”

The corrected sentence appears below:

“The third employs the Dispositional Flow Scale-2 (Jackson and Eklund, 2002; Jackson et al., 2010) for flow state data.”

The original version of this article has been updated.

